# Capacitance Technology Enables Automated Feeding, Improved Expansion, and Higher Throughput of CAR‐T Cell Stirred‐Tank Bioreactor Cultures

**DOI:** 10.1002/biot.70226

**Published:** 2026-04-20

**Authors:** Ivano Luigi Colao, Jacob Cunningham, Matthew Lee, Stephen Goldrick, Qasim Ali Rafiq

**Affiliations:** ^1^ Department of Biochemical Engineering University College London London UK; ^2^ Aber Instruments Ltd. Aberystwyth Wales UK

**Keywords:** bioprocess 4.0, cell and gene therapy, chimeric antigen receptor T cells, dielectric spectroscopy, Jurkat, process analytical technology

## Abstract

Chimeric antigen receptor (CAR) T cells present a novel and transformative approach to treat certain haematological malignancies. However, CAR‐T cell expansion methods are still under development and often rely on manual, low‐control culture methods. The transition to bioreactors would allow for greater process control and scalability and is a key focus of research in the field. Despite this, there are few methods to determine culture progression without the need for manual cell sampling which risks introducing errors and heightening contamination risks. In this article, we assessed whether capacitance technology could deliver reliable, on‐line cell concentrations by comparing T cell, and CAR‐T cell bioprocesses with Chinese hamster ovary (CHO) cultures—widely used in biotechnology and with established capacitance usage. Finding that capacitance technology could accurately measure CAR‐T cell concentrations, we then demonstrated the automation of feeding using capacitance‐derived triggers which improved bioprocess performance in terms of cell concentration and throughput. We anticipate that this study will expand avenues of investigation regarding capacitance as a suitable process analytical technology (PAT) to enable monitoring and control of CAR‐T cell manufacture, and potentially other cell and gene therapy products. It may also enable remote monitoring of multiple batches, harvest control, and the generation of large collections of process data for modelling, which will further progress the field.

## Graphical Abstract and Lay Summary

This article demonstrates how capacitance probes can be applied to single‐patient (autologous) manufacturing processes within the cell and gene therapy (CGT) industry by using CAR‐T cells as a case study. Current CGT processes require operator intervention to sample, monitor and harvest each batch which increases variability, contamination risk, and cost. By leveraging capacitance probe technology and discussing current technological limitations, we showcase how process automation could improve bioreactor culture outputs. (Created in BioRender. Colao, I. (2026) https://BioRender.com/z4mv2c1.)

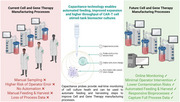

## 1 Introduction

Cell and gene therapies (CGT) are a novel class of medicines that aim to cure diseases previously considered incurable. The mechanisms of CGT products include the delivery of cells which can repopulate or restore damage caused by disease, or by the replacement, inactivation, or addition of genes [1]. Many CGT products are at the early stages of development, frequently employing manual, flask‐based culture methods. However, current research is focused on transitioning CGT candidates to processes with greater control and reproducibility, such as bioreactors [2].

One therapy which has shown clinical promise, and in which bioreactor studies have been conducted [3, 4], are chimeric antigen receptor (CAR) T cells. CAR‐T cells are an autologous product (derived from the patient, for use in the patient) for the treatment of haematological cancers [5]. The manufacturing process is intensive [6]. Beginning with patient leukapheresis, isolated T cells are activated to enable proliferation. The cells are then genetically modified via viral transduction to express the CAR antigen, prior to further expansion, purification, and secondary manufacture before re‐transfusion into patients [7].

As an autologous product, CAR‐T cell manufacture faces many challenges [8]. One challenge associated with all autologous products is that each patient requires an individual bioprocess; this creates logistical burdens and requires intensive operator organization. Additionally, CAR‐T cell processes do not currently have methods for online, real time monitoring during their expansion. Instead, culture processes rely on manual sampling to determine cell numbers, health, and to determine harvest point. This not only compounds the challenges previously described, but also increases likelihood of introducing operator error, and increasing contamination risk and potential mixing of batch data.

Adoption of online monitoring tools may alleviate some of these challenges. Online monitoring can reduce the burdens associated with operator intervention across multiple simultaneous culture processes, whilst concurrently gathering complete process datasets. These datasets may then be used to model CAR‐T cell processes (at the individual or campaign scale) to attain deeper process understanding and to facilitate adaptive manufacturing strategies. In turn, this would benefit CGT manufacturing [16] as personalized, real time culture information would streamline the decision‐making process and de‐risk cell expansion, thereby reducing the likelihood of needing to restart an already intensive and costly procedure. Tools which enable automation may also produce solutions for challenges associated with standardization across decentralized manufacturing sites, such as in CAR‐T cell manufacture [8].

This development would also coincide with wider regulatory drives for deeper process understanding by application of process analytical technologies (PAT) [9, 10], and the shift to (bio)industry 4.0 [11, 12] and smart manufacturing. As such, it is logical that the CGT sector adopts technologies commensurate with current regulatory expectations as early as possible.

**FIGURE 1:**
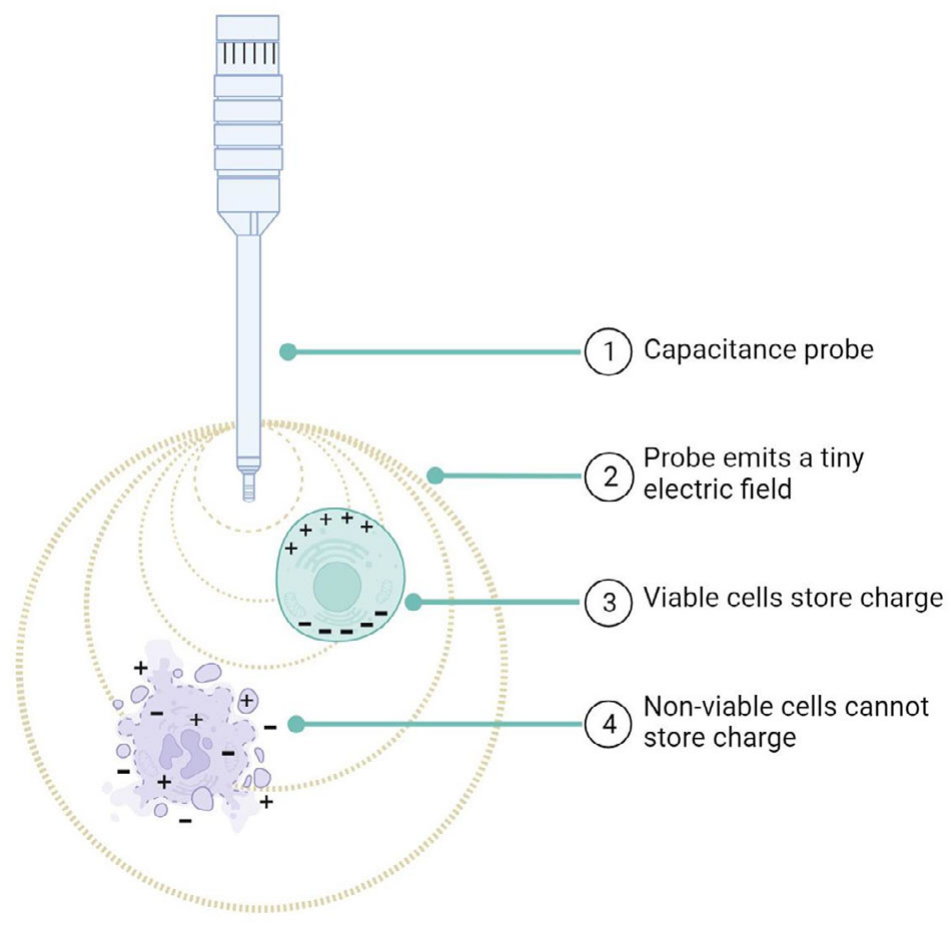
Illustration of charge storage capacity between viable cells and nonviable cells. Viable cells possess intact membranes allowing cells to act as capacitors under influence of an electric field. Nonviable cells lose this ability. Image created in Biorender.com.

Dielectric spectroscopy, commonly referred to as capacitance, is one such technology which can be applied to bioprocesses for greater process understanding [13]. The method involves the use of a probe which emits a small electric field (with negligible, if any, effect on the cells) into the cell culture. This causes charge buildup within intact cells which act like capacitors. The stored charge is proportional to the number of viable cells, thus allowing measurements of cell density [14]. This is because nonviable cells have ruptured membranes which prohibit charge accumulation [15, 16], depicted in Figure 1. The probes then record the total surrounding capacitance which gives readings proportionate with viable cell concentration [17, 18] (or more specifically, viable biovolume, as cell size can influence the magnitude of capacitance).

To date, capacitance technology has found many uses within biotechnology from brewing and fermentation [19, 20] to biopharmaceutical/therapeutic protein production [21], viral vaccine/vector production [22, 23] and even now in lab grown meat manufacture. As such, the technology has been employed widely across a variety of clinically relevant cell types ranging from microbial [24] to mammalian expression systems [25]. This inclusion in cGMP relevant processes highlights the technology's suitability for CGT application.

Capacitance technology is already established to be robust and can provide outputs such as viable cell density monitoring [20], identification of early apoptosis [25] and the detection of viral transfection and release [23], amongst many others [26]. Capacitance has also demonstrated capabilities to control feeding and cell density in fed‐batch [27, 28] and perfusion cultures [29].

Despite these successes, adoption in the CGT industry has been limited even though the technology may be readily applied to CGT processes [30]. A reason for this is that the cell and gene therapy industry is still in its infancy, with processes often using simple but unscalable formats offering little control, for example, flask‐based culture [21, 31, 32]. As these therapies approach clinical milestones, however, the need for defined and robust industrial bioprocesses increases. This will involve the transition to bioreactor cultures to generate sufficient cell yields, tighten bioprocess parameter control, and establish commercially viable methods of product scaling [7], for autologous and allogeneic [33] products.

To this end, we aim to show how capacitance technology can be a robust addition to the CGT toolkit which should be adopted from the wider biotechnology industry. Guided by this sentiment of adopting technologies from established pharmaceutical processes (which has been echoed historically [30, 34]), we hypothesized that adoption of capacitance could allow automated control of individual batches, thus enabling tailored processes for tailored medicines. We believe that this will ease the burdens associated with scaling autologous products [31], and facilitate safer, cheaper, and more robust manufacture. As the cost of CGTs is often prohibitive, technologies that facilitate reductions to the cost of goods (COGs) and improve process success/efficiency would make such therapies more accessible to healthcare providers, and thus, patients.

To prove this and owing to the higher concentrations and larger biovolumes of traditional mammalian cultures, our first aim was to demonstrate that capacitance could meaningfully measure changes in culture concentration for CGT application. To do this, we assessed the feasibility of capacitance technology to be translated from a wider biotechnology application by comparing the expected outputs of CHO culture, to those of smaller, less concentrated T cell and CAR‐T cell cultures. We also used the experiments to compare data from larger, established capacitance probes to a beta‐version, probe in development for the smaller scale bioprocesses used in autologous manufacturing. Finally, we assessed whether the measurements could automate the feeding and control of CAR‐T cell bioreactor cultures.

## 2 Materials and Methods

### 2.1 CHO Culture

CHO cells were grown in Freestyle CHO expression medium (Thermo Fisher Scientific, UK) supplemented with L‐glutamine (40 mL per liter of basal medium) (1000x stock, Gibco). Medium was pre‐warmed to 37°C prior to introduction of cells.

A cell bank of 1 × 10^7^ cells was sufficient to seed a single 125 mL vented cap shake flask containing 30 mL of fresh medium. Cell revival was performed under timed conditions, with a maximum of 5 min between submersion into the water bath to medium exchange centrifugation. Cell banks were thawed, transferred to a biological safety cabinet, and then resuspended in 9 mL of fresh, pre‐warmed growth medium. Cells were centrifuged at 400 g for 5 min and the supernatant discarded. Cell pellets were resuspended in 5 mL fresh medium and seeded into a 125 mL shake flask containing 25 mL of medium to yield a final volume of 30 mL per flask (seeding concentration was 3–5 × 10^5^ cells mL^−1^). Flasks would be returned to the humidified orbital shake platform incubator and maintained at 37°C, 8% CO_2_, 150 RPM.

Cultures would be allowed to expand until approximately 1–1.5 × 10^6^ cells mL^−1^ was achieved, at which point flask contents would be split into new flasks and subsequently diluted with fresh, pre‐warmed culture medium to seeding concentrations.

For inoculation into the bioreactor, a cell count was taken and the volume of CHO cell suspension required to provide a seeding concentration of 3 × 10^5^ cells mL^−1^ was calculated. This volume was transferred to 50 mL tubes (balanced for centrifugation). Cells were pelleted by centrifugation at 400 g for 5 min. T cells were resuspended to a total volume of 100 mL to be seeded into the bioreactor. Care was taken to ensure that cells were homogenously suspended within the seed flask and that no clumping had occurred.

### 2.2 CHO Bioreactor Operation

CHO cells were grown in Bioblu 1c single use vessels (Eppendorf, Germany). Vessel control was performed on an Eppendorf BioFlo 120 control tower. Running conditions were set to 37°C, pH 7.0, DO_2 MIN_ = 50% and 190 RPM. Base addition was automated by the BioFlo 120 tower and used 1 M sodium hydroxide. Acid titration was performed by CO_2_ sparging (automated by the tower). Oxygen level maintenance was performed by sparging air, and if insufficient, pure O_2_. Antifoam C (Sigma) was used for foam control.

Alongside a pH probe (Mettler‐Toledo), a DASGIP DO sensor (Eppendorf), and a Pt‐100 RTD (resistance temperature detector) temperature probe (Eppendorf), a Futura capacitance probe (Aber Instruments) running on Futura SCADA software was also used.

CHO cells were inoculated into the vessel via pump addition after the capacitance probe had settled and was zeroed. Cells were grown until decline phase of culture so that the end‐phase data could be recorded, at which point runs were terminated.

### 2.3 T Cell Culture

Jurkat cells were grown in complete RPMI (cRPMI). 500 mL of RPMI 1640 was supplemented with L‐glutamine (5.5 mL per 500 mL of basal medium) (1000x stock, Gibco) and 55 mL FBS (Thermo Fisher Scientific). Medium was pre‐warmed at 37°C prior to introduction of cells.

A cell bank of 5 × 10^6^ cells was sufficient to seed a single vented cap T75 flask containing 10 mL of fresh medium. Cell revival was performed under timed conditions, with a maximum of 5 min between submersion into the water bath to medium exchange centrifugation. Cell banks were thawed, transferred to a biological safety cabinet, and then resuspended in 9 mL of fresh, pre‐warmed growth medium. Cells were centrifuged at 400 g for 5 min and the supernatant discarded. Cell pellets were resuspended in 1 mL fresh medium and seeded into a T75 flask containing 9 mL of medium to yield a final volume of 10 mL per flask (seeding concentration was 3–5 × 10^5^ cells mL^−1^). Flasks were returned to the humidified incubator and maintained at 37°C, 5% CO_2_.

Initial T75 culture was allowed to expand until approximately 1–1.5 × 10^6^ cells mL^−1^, at which point flask contents would be passaged and split into Erlenmeyer flasks to original seeding concentrations. To perform the passage the cell suspension was centrifuged at 400 g and the spent medium discarded. Fresh, pre‐warmed cRPMI was added and the pellet resuspended prior to seeding into Erlenmeyer flasks containing fresh pre‐warmed medium. Cells were maintained in shake culture (150 RPM) until sufficient numbers were generated for bioreactor seeding.

### 2.4 T Cell Stirred‐Tank Bioreactor Operation

Jurkat cells were cultured in a 2.2L working volume Eppendorf glass vessel. Vessel control was performed on an Eppendorf BioFlo 120 control tower. Running conditions were set to 37°C, pH 7.0, DO_2 MIN_ = 50% and 200 RPM. Base addition was automated by the BioFlo 120 tower and used 1 M sodium hydroxide. Acid titration was performed by automated CO_2_ sparging. Oxygen level maintenance was performed by sparging air, and if insufficient, of pure O_2_. Antifoam C (Sigma) was used for foam control.

Alongside a pH probe (Mettler‐Toledo), a InPro 6860i DO sensor (Mettler‐Toledo), and a Pt‐100 RTD (resistance temperature detector) temperature probe (Eppendorf) a Futura capacitance probe (Aber Instruments) running on Futura SCADA software was used for capacitance measurements.

### 2.5 CAR‐T Cell Culture

A flask of CAR‐T cells was donated for use in this project. The CAR‐T cells were first produced per the method reported in Springuel et al. [35]. Subsequent flasks were split and expanded for 2–3 days and maintained between 0.5 × 10^6^ and 1.5–2 × 10^6^ cells mL^−1^. The original flask was banked to generate working cell banks of CAR‐T cells for the bioreactor runs. Banks were prepared by pelleting the cell suspension (400 g centrifugation, 5 min) discarding the supernatant and resuspending in CryoStor CS10 (Stem Cell Technologies). Banks were cooled at an approximate rate of −1°C per minute by use of Mr. Frostys containing isopropanol in a −80°C freezer overnight, prior to transfer into liquid nitrogen the next morning.

Upon revival, CAR‐T cells required reactivation using Dynabeads (Dynabeads Human T Activator CD3/CD28, Gibco) and Interleukin‐2 (IL‐2) (Miltenyi Biotec).IL‐2 was added in aliquots created according to the manufacturer's instructions and added to both shake flask and bioreactor culture media at 30 UI mL^−1^. As IL‐2 degrades, we assumed full degradation after 3 days of culture in both vessel and flask and we would replenish via bolus addition as appropriate for the volume of the vessels.

Dynabeads were added to both seed train flasks and bioreactors at a 1:1 ratio with the number of seeded cells. Dynabeads were prepared prior to use according to the manufacturer's instructions. Briefly, Dynabeads were triturated in the vial to disperse homogenously. The required volume was aspirated and transferred to 1.5 mL Eppendorf tubes which were subsequently placed on a magnetic separator to allow storage medium removal. Beads were washed with PBS, removed from the magnet, triturated, then replaced on the magnet for PBS removal. This was performed in triplicate, prior to resuspension of the beads in culture medium and addition to flasks (by pipette) or bioreactors (syringe injection via septum). IL‐2 addition to bioreactors was performed by sterile syringe injection via septum.

CAR‐T cells were cultured in T‐flasks kept in a humidified incubator (37°C, 5% CO_2_) for the seed culture. Culture medium was complete RPMI (cRPMI) as described for Jurkat cells. Seeding concentration was 3 × 10^5^ cells mL^−1^, splitting concentration was 1–1.5 × 10^6^ cells mL^−1^.

### 2.6 CAR‐T Cell Stirred‐Tank Bioreactor Culture

All CAR‐T cell processes were performed in Applikon MiniBio 500 mL (Getinge) glass vessels linked to My‐Control units. Vessels were automated to control for pH 7, DO_Min_ 50% and N_Max_ at 180 RPM. Impeller used was a single, stainless steel marine impeller.

To measure capacitance, beta version Pico probes (from Aber Instruments’ Futura range) were used. Assembled bioreactors were autoclaved but as the Pico probes were non‐autoclavable, they were not included in the autoclave cycle, instead being sanitized separately. As such, the PG8 fitting for the probe in the bioreactor headplate was left vacated (sealed by aluminum foil) during autoclaving. The probes were submerged in ethanol (not including the electrical connector at the top which sits outside the vessel) and stored in a biological safety cabinet overnight. Once autoclaving was complete, the vessels were sprayed liberally with ethanol and transferred to the biological safety cabinet. They were UV‐ed for 1 h to ensure surface sterility at which point the probes were introduced.

A licence was purchased from Getinge to allow direct communication between the sensor and the bioreactor controller which enabled the capacitance‐predicted biomass as a control loop set point. To obtain a predicted biomass a single 1‐point calibration was performed by drawing a sample from early‐exponential growth post‐inoculation of the bioreactor. The cell concentration recorded was obtained from the Nucleocounter NC‐250 (counting methods described under cell counting section). During the “blind CAR‐T automated” run (see: feeding regime overview) the calibration was left unchanged from the previous run's calibration settings as a way of determining how variable such measurements may be across process runs.

### 2.7 Capacitance Measurements

CHO and Jurkat scales used 1 and 2L bioreactors, respectively, which allowed for use of established reusable Futura probes (Aber Instruments). CAR‐T cell culture, however, was performed in smaller, 500 mL bioreactors and made use of beta‐version Pico capacitance probes (also, Aber instruments).

In both cases, probes were designed for equivalent performance specification which were comparable across their range of probes. Specifically, the frequency scan range was 50 kHz–20 MHz with measuring ranges from 0 to 400 pF cm^−1^, conductivity ranges from 1.0 to 40.0 mS/.cm^−1^ and a stability range of better than ±0.2 pF cm^−1^ at constant temperature across the measurement range. The signal to noise ratios of the probes are approximately the same, however a specific value is not obtainable due to variations in individual processes influencing the exact ratio.

For cell culture processes (such as in this article) the Aber system recommends a single frequency measurement mode of 580 kHz to allow the system to achieve higher capacitance signals which is crucial for accurate cell culture monitoring while avoiding undesirable influences from polarization. The system also employs a robust polarization correction algorithm to maintain accuracy at this frequency. This setting was used for all capacitance data recorded in this article.

### 2.8 Feeding Regime Overview

In this paper, Jurkat and CAR‐T cells were fed via multiple strategies. For simplicity of reference each method has been described alongside key rationale, equations, and methods. In all cases feeds were kept on ice in insulated containers to avoid degradation. Table 1 is given to summarize the major details of each run, but an in‐depth section for each run has been given to allow reproducibility, and understanding of motivation and rationale in experimental design.

**TABLE 1:** Reference table for process details. The table outlines the process details including run identifier, bioreactor type, probe used, and key feeding/process details.

Processes name	Bioreactors	Probe	Feeding regimes/Notes
CHO 1, CHO 2, CHO 3	Bioblu 1c	Futura	Batch (1L)
T Cell (Jurkat) batch	Eppendorf 2L glass vessel	Futura	Batch (1.2L)
Lab‐based bolus (Jurkat/T cell)	Eppendorf 2L glass vessel	Futura	Initial volume = 1.2L Bolus 1 (0.2L) added at 72 h Bolus 2 (0.3L) added at 96 h Bolus 3 (0.3L) added at 120 h
Capacitance‐based bolus (Jurkat/T cell)	Eppendorf 2L glass vessel	Futura	Initial volume = 1.2L Bolus 1 (0.2L) added @ C = 2 pF cm^−1^ Bolus 2 (0.4L) added @ C = 2 pF cm^−1^ Bolus 3 (0.4L) added @ C = 2 pF cm^−1^ (± 0.1 pF cm^−1^ tolerance on feed initiation)
Linear feed (Jurkat/T cell)	Eppendorf 2L glass vessel	Futura	Initial volume = 1.2L Feed triggered at 1 pF cm^−1^ (1L added over 72‐h period)
Exponential feed (Jurkat/T cell)	Eppendorf 2L glass vessel	Futura	Initial volume = 1.2L Feed triggered at 1 pF cm^−1^ Feed rate of 42 mL h^−1^ (rate approximately matches exponential growth rate of cells)
**For all CAR‐T cell processes (below) IL‐2 was added every 3 days (2 µL per mL culture volume at time of addition)**			
CAR‐T batch 1 CAR‐T batch 2	Applikon MiniBio	Pico	Batch (280 mL)
CAR‐T manual feed	Applikon MiniBio	Pico	Initial volume = 260 mL Feed triggered manually at 9 × 10^5^ cells mL^−1^ 200 mL feed added Feed rate of 9.86 mL h^−1^ (rate approximately matches exponential growth rate of cells)
CAR‐T automated feed	Applikon MiniBio	Pico	Initial volume = 260 mL Feed triggered by automated control loop set at 9 × 10^5^ cells mL^−1^ (as determined by calibrated biomass) Feeding triggered similar to pH or DO control loops to maintain constant (cell) concentration
CAR‐T Blind manual feed	Applikon MiniBio	Pico	Initial Volume = 260 mL Feed triggered at C = 1.1 pF cm^−1^ (hypothesized to represent 9 × 10^5^ cells mL^−1^, hence “blind”) 200 mL feed added Feed rate of 9.86 mL h^−1^ (rate approximately matches exponential growth rate of cells)
CAR‐T blind automated feed	Applikon MiniBio	Pico	Initial volume = 260 mL Feed triggered by automated control loop set at 9 × 10^5^ cells mL^−1^ (as determined by calibrated biomass: calibration used the data from previous run, hence “blind”) Feeding triggered similar to pH or DO control loops to maintain constant (cell) concentration

*Lab‐based bolus (Jurkat/T cell)*. The laboratory‐based bolus is named for the usage of similar feeding strategies in early transitional experiments from flask to bioreactor culture. Feeding occurs in disparate boli, in our case, triggered every 24 h on Days 3, 4, and 5. To perform this, a media bottle was marked with the necessary increments (from top down) and a pump manually switched on for each volume required, that is, 200, 300, and 300 mL.

*Capacitance‐based bolus (Jurkat/T cell)*. The capacitance‐based bolus is named for triggering bolus addition based on the capacitance values, rather than an arbitrary schedule‐based 24‐h setting. Whilst this may prove inconvenient for manual operation the end goal of this study was automation. When capacitance measurements stably reached 2 pF cm^−1^ (±0.1 pF cm^−1^) the first bolus (200 mL) was triggered. In response to cell dilution, capacitance would fall. When 2 pF cm^−1^ was achieved again, the second bolus would be added (400 mL), and likewise repeated for the third bolus (400 mL). Should capacitance fail to reach 2 ± 0.1 pF cm^−1^ at any point, no bolus addition would be made, and the run allowed to reach failure. This would demonstrate a lack of applicability in the value selected and would have driven further experiments to select a more appropriate point.

To feed at each value of 2 pF cm^−1^ the run's real time capacitance values were used. Using the rationale that cell concentration is proportional to capacitance in exponential growth, the operator would use the in‐progress dataset to generate growth curves utilizing every 120^th^ value (corresponding to approximately 1 h of readings) to generate manageable cell growth data for rapid processing. From this, a curve would be fitted with an exponential line of best fit, where the exponent equates to the cell growth constant. Using this constant, one could predict the trajectory of the graph to estimate the time to reach 2 pF cm^−1^.

This worked reasonably well although—for convenience to others who wish to replicate such a study—there is some limitation to this method. Due to the nature of such feeds exhausting the cells and nutrient depletion, growth was not constant, thus it was often a few hours after the prediction that the feed was required to be triggered.

*Linear feed (Jurkat/T cell)*. This process fed an additional 1 L of culture medium over the same 3 days as the lab‐based bolus (LBB), but instead by continuous feeding at a fixed flowrate (i.e., the average flowrate for 1 L over 72 h). This experiment was designed to show how continuous feeding avoids the deceleration/reacceleration of cell metabolism/growth which is caused by bolus additions.

*Exponential feed (Jurkat/T cell)*. Like the linear feed, this regime used continuous feeding. However, the rate was matched to approximate the exponential increase in cell concentration over time during the exponential phase. Knowing that capacitance (during exponential growth) can model viable cell measurements, a value of the cell growth constant was obtained (by either semi‐log plot, or exponential curve fitting to the dataset capacitance versus culture time). Using the cell growth constant, we then performed a calculation of bioreactor volume expansion. That is:


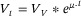

where, *V*_V_ is the original vessel working volume (1200 mL), *V_t_* is the predicated vessel volume at time *t*, *e* is Euler's number, *μ* is the derived cell growth constant (0.0340 h^−1^), and *t* is elapsed culture time (h) from hypothetical feed trigger.

By using a range of t values from 0 to 30 h one can model how the volume of a bioreactor would need to increase over time to match cell growth (i.e., to maintain constant concentration). Values above the maximum working volume are obviously ignored. Here, maximum working volume was achieved in 18 h. Using this value as the time for feeding, and the volume of the feed available (1 L) we calculated the average feed rate to approximate the growth kinetics. This yielded a requirement for 55 mL h^−1^. As pump settings could not achieve this exactly, the closest value was selected resulting in feeding of 42 mL h^−1^ over a period of 24 h. Use of a controller to maintain C as a constant value is a natural progression to this in automated cultures.

*Automated and manual CAR‐T*. The CAR‐T cell batch data were analyzed retrospectively for the values of cell growth constant over the culture period (as described previously). This yielded the results shown in Table 2.

**TABLE 2:** Cell growth constant over a CAR‐T cell batch. The table lists calculated cell growth constants during a CAR‐T cell batch process, used to generate feeding strategy. Maximum value of cell growth constant (*μ*) observed was 0.0301 h−1.

Time (h)	*μ* (h^−1^)
60	0.0278
50	0.0296
40	0.0301
30	0.0301
20	0.0251

From these values we performed the same feeding approximation as described in exponential feed (Jurkat) for the manual control. Using the historic data, the time where 0.0301 h^−1^ was observed (i.e., 30–40 h) gave us the corresponding cell concentration with which to set the Applikon biomass controller for the automated run, in this case the values corresponded to a concentration of 8 × 10^5^–1 × 10^6^ cells mL^−1^. The mid‐point 9 × 10^5^ cells mL^−1^ was selected to trigger feeds. This was expected to equate to capacitance values of 1.1–1.5 pF cm^−1^.

For automated control the Applikon MiniBio systems required in‐process calibrations which could convert the capacitance signal into the cell concentration for automation. To do this, a sample was taken prior to where feeding would be expected to begin (the pre‐selected 30–40 h) and a viable cell concentration value was calibrated to the capacitance measurements. To avoid issues with this first use, the control loop was only turned on when the values were calibrated, and 5 min had passed to ensure stability (in case calibration was taken at a fluctuation point for example).

*Blind automated and blind manual CAR‐T*. Conditions were identical to those in the automated and manual CAR‐T cell runs described above. However, samples could not influence the triggers of the runs. For the automated run, the control loop was turned on immediately upon process start using the calibration data from the previous automated control run (Automated CAR‐T). This meant aberrant/premature feeding could be identified if it occurred (and would have resulted in run failure). For the manual run, the capacitance value of 1.1 pF cm^−1^ was given as the trigger (based on previous observation) for feeding, which in turn would correspond to the desired values of *μ* (0.0301), viable cell concentration (9 × 10^5^ cells mL^−1^).

Doing this experiment allowed us to prove the applicability of the technology to automate feeding and accurately estimate cell concentration. By having derived cell kinetic constants to generate feeding regime data in the manual runs, we showed how models/estimates can be made with little previous information in proof‐of‐concept evaluations for studies where advanced controllers may not be readily obtainable.

### 2.9 Cell Counting

*Nexcelom cellometer Auto 2000*. For Jurkat and CHO experiments the Nexcelom Cellometer Auto 2000 was used to count cells. Bioreactor samples would be taken by discarding 1.5x the dead volume of the sample port/line prior to collection of fresh samples. Cells would be vortexed briefly to homogenously suspend and a 20 µL volume transferred to an Eppendorf tube, ensuring that the volume was placed directly at the bottom of the vial. To this, 20 µL of trypan blue was added, and mixed. 20 µL of this solution was immediately transferred to the cell counter counting chambers and processed. A second sample was then processed in the same way using the original sample vial (revortexed).

*Nucleocounter NC‐250 (CAR‐T cells)*. A nucleocounter NC‐250 (Chemometec) was used for CAR‐T cell counting. Bioreactor samples were taken by discarding 1.5× the volume of the sample port, prior to removing a 1.5 mL sample. This was transferred to an Eppendorf tube which was then vortexed. The NC‐250 cell counting cassettes were then submerged in the sample and used to aspirate the required volume of homogenous cell suspension (controlled by the cassette itself). This was then placed into the nucleocounter which automated the acquisition and counting of cells. Again, cell counts were performed in duplicate.

*Cell revival and sub‐cultures*. A revival timer was set to 5 min. In this time cells would be required to have been defrosted in the water bath and placed into the suspension medium for centrifugation.

*Inoculation and process timer*. Capacitance probes perform best when they have had time to settle prior to being zero‐ed (especially if the vessel is still reaching set point conditions). As such, operation of all probe/processes required zero‐point standardization. To do this, probes would be turned on once bioreactors had been conditioned and were ready for inoculation. Once process conditions were settled, probe readings would be checked for stability. When stable, the probe would be zeroed. At this point cell inoculum could be prepared. If the probes remained stable at zero, the inoculum could be introduced, and the process timer begun.

This allowed recording of the exact start time to allow easy data handling between the probes/process data and manual samples. The bioreactor laptop was always used for time‐point acquisition. Start time always corresponds to the beginning of inoculation.

As capacitance requires approx. 15 min to stabilize post‐inoculation, the first process sample was taken after 15–30 min to allow the manually recorded capacitance value at time of sample to be accurate. In graphed data these first samples would be given a non‐zero time corresponding to the time between official “start” during inoculation, and the first sample. We note this as handling multiple large data files and manually recording could easily introduce errors, by doing this we remove the risk of mis‐timings across data sets.

### 2.10 Software and Data Analysis Packages

Cell counting data were recorded and initially processed on Microsoft Excel. Origin Pro 2023 was used to produce graphs, and to perform statistical analyses and line fitting. All capacitance probes had data collected by use of Futura SCADA software (Aber Instruments). BioRender was used to create Figure 1 and the graphical abstract (Colao, I. (2026) https://BioRender.com/z4mv2c1) with permission to publish granted under the academic license.

## 3 Results

### 3.1 Current Uses of Capacitance Technology

**FIGURE 2:**
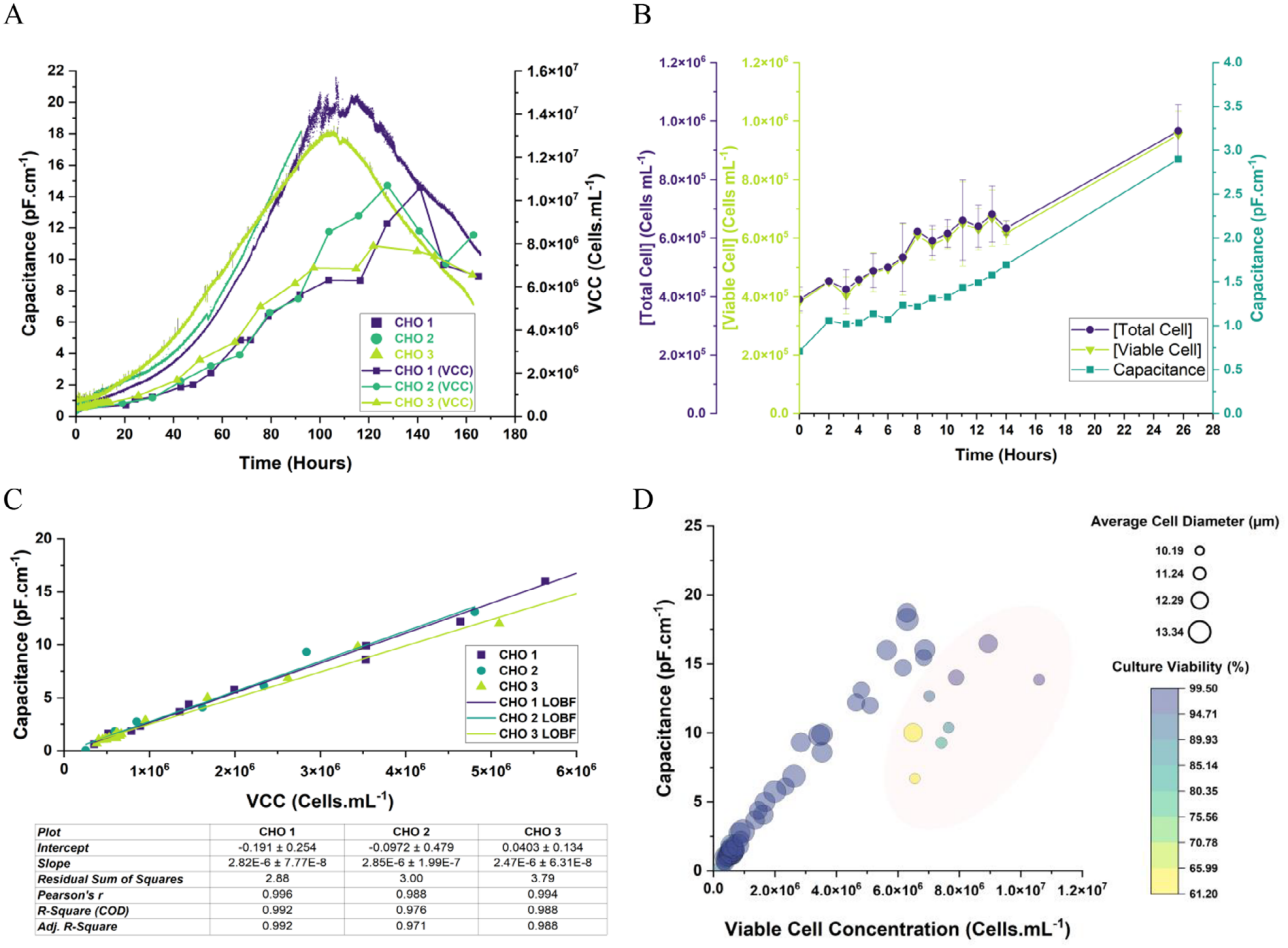
Assessment of capacitance probes for a typical biopharmaceutical CHO culture process. Panel (A) depicts a triplicate of CHO batch processes to compare the outputs of manual cell counting (scatter and line plots) and online capacitance measurement (scatter only trends). Panel (B) shows the relationship during the early and exponential phases of the CHO 3 process to highlight the lowest expected capacitance values found in current biopharmaceutical manufacturing. Panel (C) displays the linear relationship between capacitance output and viable cell counts. The panel intentionally omits the late‐stage data where viability loss and cell shrinkage skew the linearity of the trend to highlight the potential use in high viability CGT culture. Panel (D) further depicts the trend from all three batches in a bubble‐plot with cell size and culture viability as added metrics. Here the previously omitted end‐point data is highlighted to show how the trend becomes skewed.

Due to the nascent stage of CGT manufacture, we commenced our work with a traditional, biotechnology system: Chinese hamster ovary (CHO) cells. Doing so allowed us to create a logical start‐point with which to test our initial hypotheses. In Figure 2 we show a triplicate of CHO cultures to outline the background upon which this study was based.

Figure 2A shows the results of the three CHO batches. The data show that the increase in viable cell density corresponds, from inoculation to cell decline, to increases in capacitance. As cell concentration increases, a greater capacity to store charge induced by the probes is possible. The capacitance trend grouping is tightly conserved during the triplicate runs, as is the correlation between cell counts (which are also reproducible between runs) suggesting the probes are accurate and precise in their measurements. Of importance is the scale of the axis: the capacitance values are shown to initiate (in Figure 2B which highlights samples from the initial 26 h of culture) at approximately 0.75 pF cm^−1^ from an inoculation concentration of 2 × 10^5^ cells mL^−1^. This is a key detail as CHO cells are both larger and more densely growing than T‐ and CAR‐T cells; thus a main concern of the study at initiation was the ability of the probes to detect a range of capacitance values which would likely be far lower than the typical 1–20 pF cm^−1^ range observed in industrial cultures.

Figure 2C, which uses only early‐exponential phase data from the runs, illustrates the ability of the probes to give strong correlations between viable cell density and capacitance values prior to initiation of cell death. During these latter phases, the trends are shown to become erratic and divergent from the viable cell count. This is attributed to changes to cell morphology, lysis/apoptosis, and cell death‐induced medium conductivity fluctuations during the end phase and has been previously documented [36]. Such trends are also evidenced during the plateaus of CHO 1 and 3 when referring to Figure 2A.

The bubble‐plot in Figure 2D reinforces how the same correlative relationship breaks down at latter stages of culture (by re‐inclusion of the late phase data) with less viable, shrinking cell populations beginning to skew the otherwise linear fit between viable biomass and capacitance. This skew is emphasized by the highlighted region in the bubble‐plot. Such trends are crucial as the major aim of CGT processes is delivering viable cells. Thus, methods capable of providing indicators to changes in culture quality are important considerations when innovating CGT process control strategies.

From this baseline we determined a set of initial questions to be asked of capacitance technology for CAR‐T cell applicability:

1. Can the probes monitor lower concentrations (expected between 1 and 5 pF cm^−1^ due to the lower concentration and size of T cells) as accurately as they do for the larger signal runs for which they were designed?
2. Can the probes appropriately detect inoculation densities, or will there be a delay/limitation in their usage to latter stages?
3. Will the same linearity of fit still be evidenced in lower density/smaller diameter cell cultures?

### 3.2 Progressing From CHO to T Cell

To answer these questions, we progressed to cultures of Jurkat cells. Jurkat cells are an immortal T cell line which was originally isolated from a 14‐year‐old donor with acute lymphoblastic leukaemia (ALL) and are used widely in research [37, 38]. The reason for choosing Jurkat cells was partly ethical (no need to procure fresh blood for the isolation of primary T cells for a conceptual experiment) and partly practical as using an immortalized line allowed for assessment of only the probe's functionality (by removing the variability associated with donor cells which may or may not grow optimally).

To begin, we first evaluated a fed‐batch regime which is common through the literature in CAR‐T cell processing. We performed a bolus feed on Days 3, 4, and 5. This feeding regime is used as a makeshift way of transitioning from flask‐based culture where passaging/feeding of cells is often performed every 3–4 days. Doing this allowed us to evaluate the potential use in CGT‐relevant processes whilst concomitantly seeing the response of the probes with respect to feeding in order to validate the expectation of proportional drops in capacitance upon medium addition.

**FIGURE 3:**
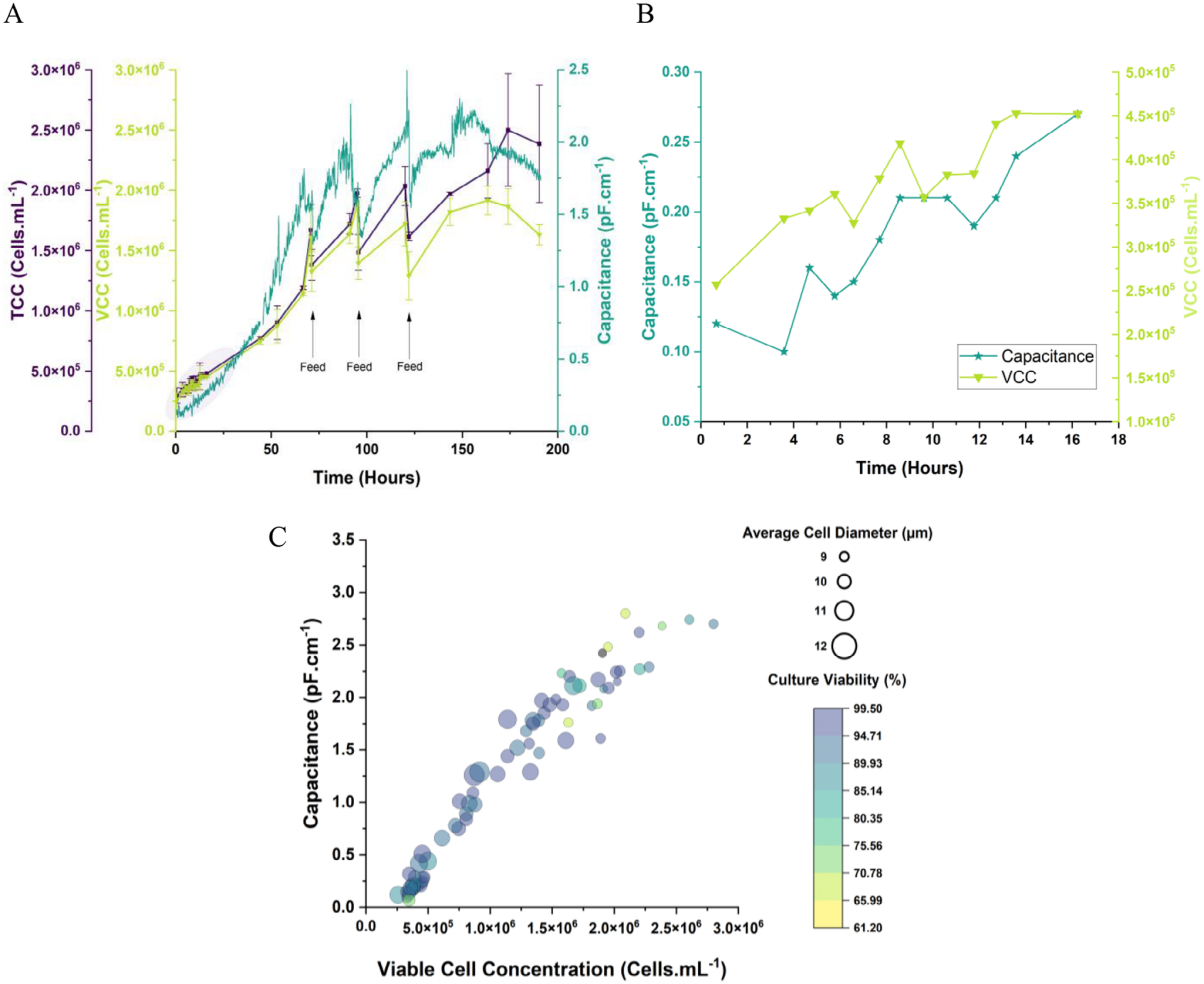
Outputs of capacitance measurements for a T cell (Jurkat) bolus fed process. Panel (A) overlays the online capacitance trace for the LBB process with the viable and total cell concentrations. Feed points have also been marked on the figure. Panel (B) reproduces the lag phase data (the highlighted region in A) for the run to demonstrate the probe's ability to produce correlative results well below the typically observed operational ranges of the probes seen in current biotechnology processes. Panel (C) is a bubble‐plot of viable cell concentration versus capacitance which displays cell diameter and overall culture viability.

Figure 3 summarizes the results gained from boli fed Jurkat culture (later referred to as the “LBB”). The figure demonstrates that despite having smaller cells at a lower concentration, the capacitance trend matches off‐line measurements of cell concentration, similar to the trends observed in the CHO experiments. Specifically, Panels **3A** and **3B** overlay the off‐line cell counts with capacitance to show proportional overlaps, whilst Panel **3C** builds on this idea by including viability data. As with the CHO results, the same skew is noted at the latter stages of culture where cells begin to lose viability, but otherwise there is strong linearity between cell concentration and capacitance readouts. This data gave us confidence that capacitance probes can tolerate the lower signal cultures for CGT applications.

Additionally, we observed feeding‐based dilutions in the capacitance data, which indicated that the probes could operate sensitively and precisely at the lower limits of their tolerance (and well below guideline ranges suggested for biopharmaceutical use). We expanded on this by showing how samples taken over the initial 18 h of culture still form a positive correlation between capacitance and the minor increases in cell concentration observed (Figure 3B). Finally, Figure 3C displays the general linearity between capacitance and viable biomass (as a function of cell size) that was observed in CHO cells, but now between the lower operational range of 0–3 pF cm^−1^. It should be noted that the results presented in **3C** are comprised of data from all the Jurkat experiments, including those presented later, but given here for logical flow: initially the data used for these observations was purely from the LBB already reported.

From this work we were able to expand our experiment list once more to something more closely related to future CAR‐T cell manufacturing processes. At this stage we now sought to determine:

1. Can capacitance technology identify trigger points for cell feeding to avoid the decline of growth observed during latter stages of bolus feeding?
2. Can continuous feeds improve CGT culture performance in line with more traditional biotechnology processes?
3. Can the use of capacitance data aid in process optimization?

### 3.3 Optimizing T Cell Feeds Using Capacitance

**FIGURE 4:**
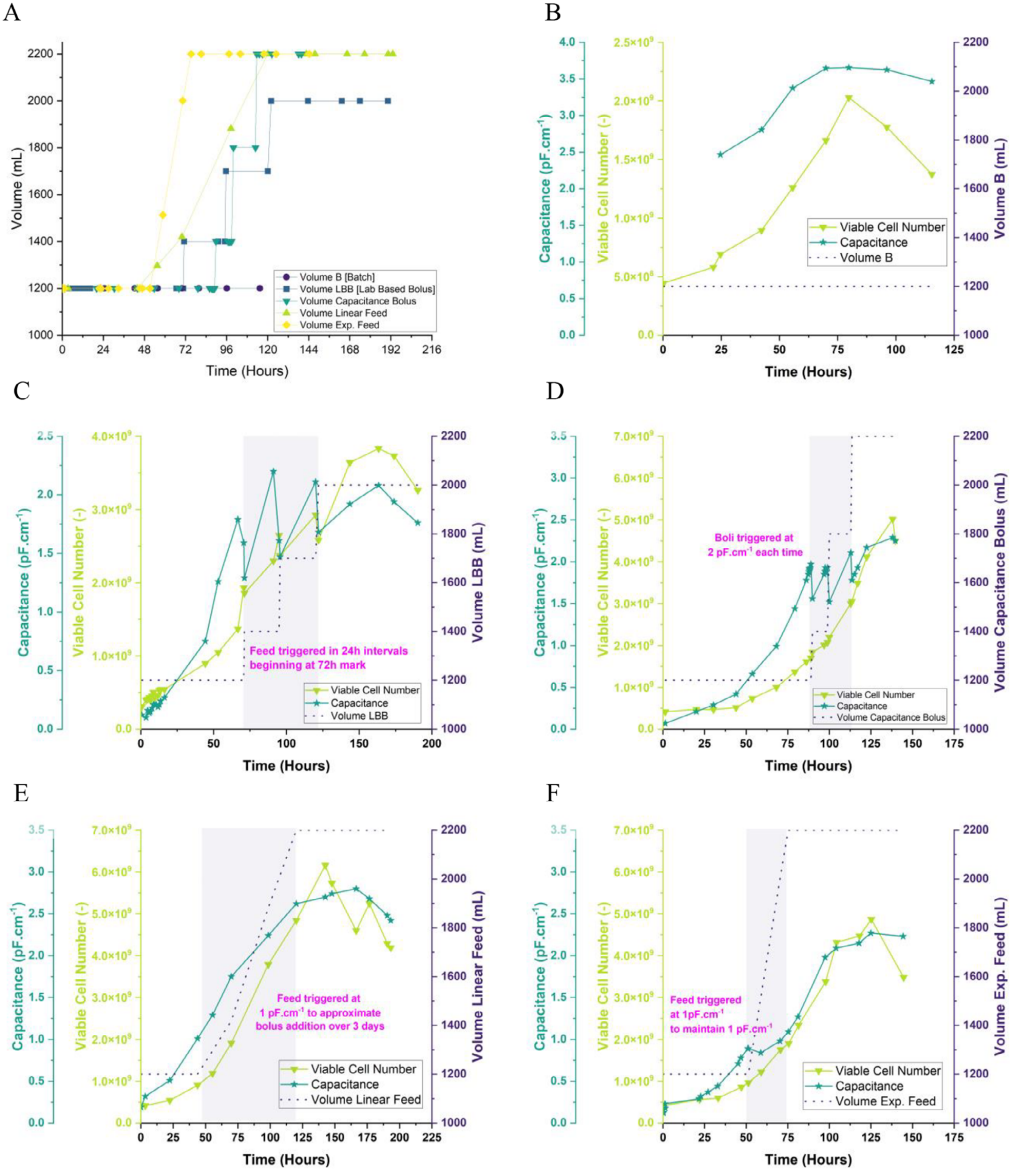
Summary data for various feeding regimes employed in T cell (Jurkat) cultures. Panel (A) summarizes the different feeding regimes with respect to process time used for the various Jurkat runs. Panels B‐F all present the graphs of sample time recorded capacitance, viable cell number and culture volume (for ease of understanding feeding implications). Feed zones are marked by the shaded region and relevant detail given in magenta text. Panel (B) = batch process; Panel (C) = LBB; Panel (D) = capacitance‐based bolus; Panel (E) = linear feed; Panel (F) = exponential Feed.

To answer the questions from the previous section, we performed a variety of fed‐batch processes. Presented in Figure 4 alongside the feeding summaries (Panel **4A**) are the results of a batch process (**4B**), the LBB (Panel **4C**), a capacitance‐based bolus (**4D**), a linear feed (**4E**) and an exponential feed (**4F**). It should be noted that the capacitance‐based runs were performed as their own set of experiments and thus the final volume of the runs are all 2.2L (as opposed to the 2L LBB) to allow the feeding durations to be extended where the continuous feeds were running. All other conditions from seeding density, freshness of inoculum trains, bioreactor settings and media compositions are identical.

With the batch run we saw that cells exhausted the medium after 80 h, in line with the feeding regimes in flask‐based culture. The maximum yield of the batch was approximately 2 × 10^9^ viable cells. By extension, we observed that during the LBB process, cell density increases upon addition of feeds at Day 3, 4, and 5. However, the growth profile also exhibits slowdown (as determined by the gradual decline in curve gradient) with each progressive feed. This suggests that the timings between feeds are sub‐optimal for maintaining an excess of culture nutrients and that, consequently, cells are undergoing fluctuations of feeding and starvation, thus impairing the efficiency of culture. Such consequences may hamper production of therapeutic CAR‐T cells especially as donor‐to‐donor variability will guarantee that such a generic, operator timing‐based protocol will not be ideal for inequivalent starting materials.

To remedy this situation we then evaluated a bolus based on the use of capacitance instead. To do this, feeds were triggered manually at the value of 2 ± 0.1 pF cm^−1^. The rationale for this value was to allow some level of comparability with the LBB run whose pre‐determined 72‐h initiation point corresponded with approximately 2 pF cm^−1^ whilst subsequent feeds were given at values of increasingly higher capacitances due to the set 24‐h feed interval (which were observed to be “late” as a consequence of triggering on schedule timing rather than a biologically valid set‐point).

From the results we observed that the viable cell count (VCC) maximum was increased from 4 × 10^9^ cells in the LBB to 5 × 10^9^ cells in the capacitance‐based variant. This is because the cells were not allowed to re‐exhaust the feed between each bolus, suggesting that finding an optimal feed point based on capacitance values (and trendline gradient analysis) could lead to immediate improvements in culture performance where a generic process is currently used. Additionally, the time to complete feeding (i.e., to reach maximum vessel capacity, and thus harvest) was shortened by 12 h, suggesting that time was being wasted by inefficient inactivity during the LBB.

The next two experiments were designed to make greater use of capacitance in terms of designing more industrially relevant feed methods.

The first of the runs, termed linear feed, matched the feeding rate to the equivalent addition of medium from the original bolus experiment over 3 days, to negate the acceleration‐deceleration phases which come from repeated bolus exhaustion between feeds. This was hypothesized to be a natural improvement to any form of bolus addition as is commensurate with standard theories of bioprocessing. The feed was triggered at 1 ± 0.1 pF cm^−1^ to show ability of capacitance to function as a surrogate for manual cell counts for feeding initiation. This value was chosen as the previously used 2 pF cm^−1^/72‐h feed triggers were based on cells completely exhausting media components. Feeding before this occurred, during mid‐exponential growth, would allow for any potential slowdowns to be reduced.

The results of the linear feed once more improved upon the bolus processes, now achieving a maximum of over 6 × 10^9^ cells, whilst still achieving feeding completion in line with the LBB process. As the feed was set to match the LBB, we posited that a faster addition rate which matches the average capacitance bolus feed might provide a more efficient process.

For the final variant, we desired to assess the overlaps in cell and capacitance data. Logically, if the increase between exponentially growing cells and capacitance increases is proportional, this must mean that all capacitance trends can be used to derive cell growth kinetics. We used the values of the cell growth constant from previous data to provide the expectation for the rate of increase of cells during the feeding period (also triggering at 1 ± 0.1 pF cm^−1^). The previous data showed a maximum growth constant (*μ*) of 0.0340 h^−1^, obtainable by either fitting exponential trendlines to the data or by the semi‐log plot method.

From this we calculated the addition rate of medium based on the value of the cell growth constant such that the volume of the bioreactor would expand in line with the increases in cell density, with the aim of maintaining a stable cell concentration during the feeding period. As we were unable to exponentially increase the feeding rate via the controls we had, we averaged the increase over time to provide a constant rate feed until 2.2L was achieved. The value of *μ* corresponded to an equivalent vessel volume increase of ca. 42 mL h^−1^ (the closest approximation from the pump settings available).

The rationale for this experiment was in part due to the desire to not flood the cells, and to maintain a homogenous, optimal concentration of cells/limited concentration of metabolites, but also to show how application of capacitance readings could be used to control advanced processes (e.g., perfusion) in future CAR‐T cell processes. Full details of rationale/set ups are given in materials and methods.

The results of this final run showed promise in using capacitance derived kinetics to maintain steady cell concentrations. This is depicted by the approximate horizontality of the trend line during the feed period. The horizontality was imperfect as a consequence of the experiment being unable to change feed rates exponentially which forced a linear approximation to the cell concentration increase over a set period. We hypothesized from this, that results may be optimized if a controller could attain such feed rate changes.

In addition, the results once more showed improvement in terms of feed completion and overall cell yield (5 × 10^9^ cells) when compared to the LBB.

Overall, the results showed that capacitance‐aided runs provided higher concentrations of viable cells than the bolus‐based methods prior to exhaustion of the cultures. Additionally, we evidenced how cell culture improvements can be achieved in a few simple steps when deeper process understanding/monitoring is applied. The linear feed performed the best in terms of total cell yield, whilst the exponential feed showed promise in the application of capacitance‐based feeds/maintenance of cell concentration in CGT relevant processes, as evidenced by the flattening of both VCC and capacitance curves during the feeding time. This approximate flattening also proved the hypothesis of transferrable growth kinetic derivation by using capacitance as a surrogate for viable cell concentration.

**FIGURE 5:**
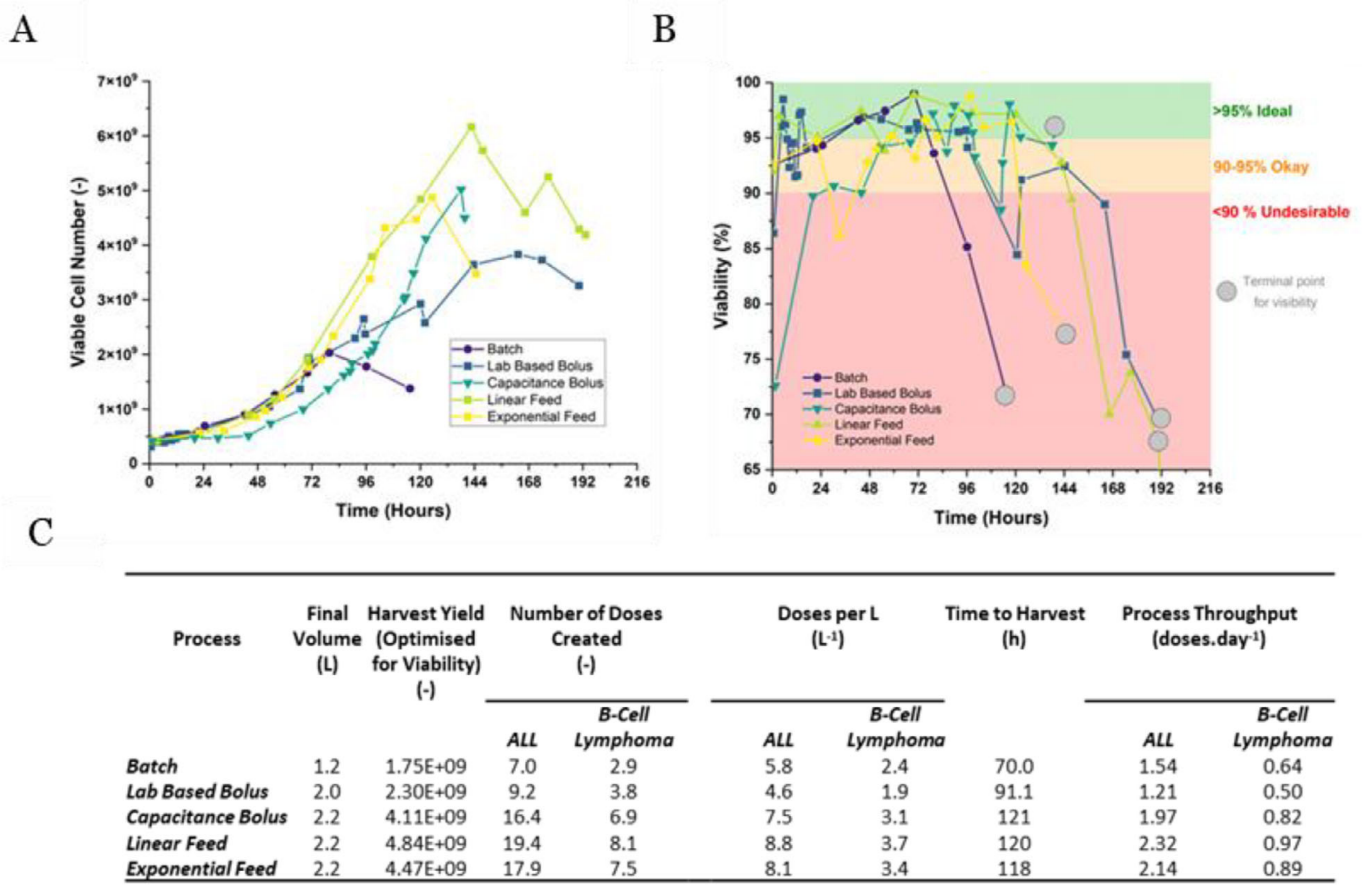
Summary data for the different feed regime processes for T cells (Jurkat). Panel (A) gives an overlaid viable cell number graph for the different processes to compare culture time and cell yields obtained across the processes. Panel (B) presents the viability data for the same processes to allow for understanding of where ideal harvest points may occur. Panel (C) contextualizes the harvest point yields (selected as last point of ideal viability) in terms of largest doses of Kymriah for the treatment of either ALL or B‐cell lymphoma.

In Figure 5 we show the summary data for the experiments to give better contextualization to the results. In Panel **5A** we show how the viable cell yields compare directly on a single scale axis. The capacitance bolus, linear feed and exponential feeds outperform the previously described lab‐based addition. Notably, the capacitance bolus also shows extended lag with the exponential phase taking longer to initiate, suggesting performance here was closer to the two continuous feeds than previously anticipated. However, viable cell count is not the only metric necessary for a therapeutic cell product. Culture viability must be maintained to clinical levels, and thus we presented a plot of viability across each culture (Panel **5B**).

Typically, biopharmaceutical cell cultures are ideally maintained around 85%–95% viability during production phases [39] to minimize the impacts of host cell proteins from entering the product stream, however for product viability in CGT applications, cells of >80% can be considered sufficient as exemplified by Kymriah [40]. For our analyses we opted for the more conservative value range of 90%–95% as the ideal threshold—partly because it favors more stringent manufacturing processes, and partly as while we're showing a case study on CAR‐T cells, we do believe that for the wider field of cell and gene therapies, picking a lower acceptance range may not reflect all cell types, or may incur unnecessary final product titre losses if the cells drastically decline. Regardless of range chosen for this evaluation though, viability criteria is an important consideration as harvest points across the CGT field will be influenced by a product's CQAs and registered release criteria.

The graph in **5B** shows that high viability is maintained post‐inoculation adaption in each culture until medium exhaustion occurs at which point the cells begin to die. For ease of following trendlines, the last point in each graph is highlighted so tracing lines can be made simpler.

The runs were allowed to reach decline and death phases for the purposes of robust data collection, and thus presentation of viable cells does not give the full picture of how each run performed. Using the viability data however, we identified harvest points for each curve prior to cellular decline. Using these values we generated a summary table (Panel **5C**) which shows the differences each method had in terms of doses of Kymriah for ALL and B‐cell lymphoma (up to 2.5 × 10^8^ CAR‐T cell positive viable T‐cells for patients >50 kg for ALL, and up to 6 × 10^8^ cells for B‐cell lymphoma as obtained from the Kymriah package insert) [41]. We saw that by using the feeds developed by capacitance information, we could immediately create methods of feeding which drastically increase the doses achieved, whilst minimizing the extra time to do so. The table in **5C** shows the relative doses per liter of each process. Despite including feeding, we observe that at the chosen harvest point the LBB is producing a lower cell per liter output than the batch run (e.g., 4.6 doses to treat ALL versus 5.8 in the batch). This suggests that using sub‐optimal feeding based on scheduling is reducing the potential of achieving higher cell recovery. When capacitance is used to trigger feeds however, the doses per liter are greatly improved, yielding between 6.9 and 8.1 doses per liter (compared to 4.6 doses per liter for the LBB and 5.2 doses per L in the batch) proving that cells maintained properly can grow more efficiently.

This is notable as autologous processing often means suites of manifold, simultaneous processes. By increasing yields to provide multiple doses (Kymriah for example is a cryopreserved product which can be stored) we can limit the need for multiple donor biopsies in the event of product damage/loss, reinfusion necessity or re‐administration/larger dose requirements. Each of these potential benefits may contribute to higher quality products and the potential to maximize the chances of success in therapeutic production without the need for multiple biopsies.

### 3.4 Capacitance Measurements in CAR‐T Cell Culture

Having shown that capacitance can be used in T cell cultures, we moved onto the proof‐of‐concept studies using CAR‐T cells. As CAR‐T cell cultures are far more limited in scalability, the bioreactor used was also changed to 500 mL Applikon MiniBio reactors which could integrate with Aber software for control experiments. We substituted the probes from the larger, established Futura probes (which were too big for the vessel) to beta‐version Pico probes (both, products of Aber Instruments) designed for use in small scale (and potentially single‐use, or CGT relevant) cultures.

**FIGURE 6:**
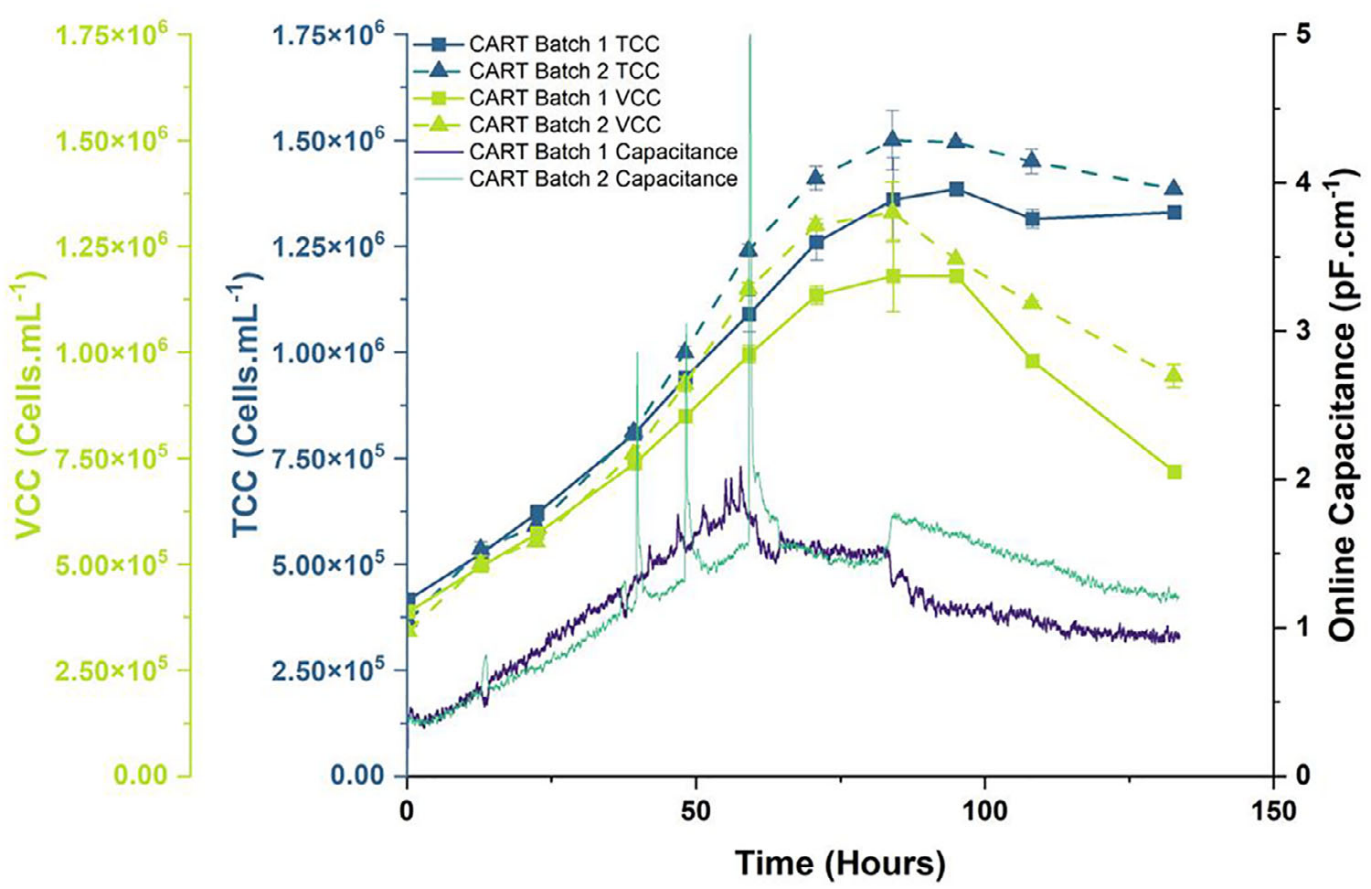
Comparison of two CAR‐T cell batch cultures assessed for total and viable cell counts, and online capacitance measurements. The graph depicts the results from two batch cultures of CAR‐T cells. Manual cell counts are depicted with squares and a solid line for batch 1, and triangles and a dashed line for batch 2 with respective trend colors matching the appropriate axes. Online capacitance trends are given as distinctive colors: purple for batch 1, green for batch 2, and are linked to the right‐hand capacitance axis.

To validate the new probes and the CAR‐T cell expectation matching to previous data, we performed two batches of CAR‐T cell cultures with the data shown in Figure 6.

The graph shows that the CAR‐T cells banked for the project were reproducible in their growth profiles, and that the probe data could adequately increase with cell concentration during the early and exponential phases of growth. We also achieved a maximum concentration of around ≈1.25–1.5 × 10^6^ cells mL^−1^ by Day 3 in both runs prior to media exhaustion and decline, echoing the expectations obtained from the Jurkat experiments.

Once more we observed the expected capacitance decline correlated with cell shrinkage/loss of viable biovolume, and an increase in noise as cells reached the latter stages of culture. Generally, the new probes performed well, although three large signal spikes were observed during the exponential phase of CAR‐T batch 2. The reason for this was unknown, however as the project aim was to ultimately demonstrate the potential of capacitance to automate CAR‐T cell culture, such artefacts could pose a problem with undesired pre‐triggering of future control strategies which is discussed further later (see: CAR‐T cell bioreactor automated control studies).

**FIGURE 7:**
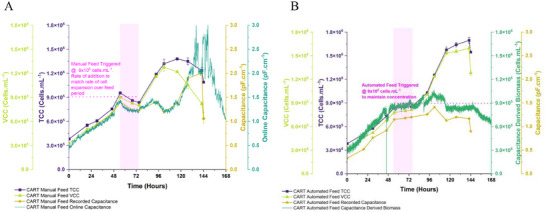
Output data for CAR‐T cell cultures with feeds based on capacitance derived information. Panel (A) represents the manual run which attempted to match cell growth rates derived from capacitance data to a linearized feed triggered when 9 × 10^5^ cells mL^−1^ was achieved. Panel (B) represents the automated feed which was triggered when the Applikon system detected 9 × 10^5^ cells mL^−1^ were present by using integrated capacitance software.

From these batches we then used the capacitance and cell data to generate new feeding regimes for the CAR‐T cells, shown in Figure 7.

Of the two bioreactors available, one was set up to be triggered manually whilst the other was installed with software allowing for automated control of the feeding based directly on the capacitance values. These experiments are referred to as manual and automated, respectively.

The manual feed emulated the exponential feeding from the Jurkat experiment to highlight how manual control guided by capacitance and cell growth data can be easily achieved even without advanced control software. The trigger was set to 9 × 10^5^ cells mL^−1^ which is where cell growth constant was highest during the batch cultures.

The automated feed, which converts capacitance directly to biomass based on an early, in‐process, one‐point calibration with a manual cell count, (and which can also be saved for subsequent runs) was identically set to maintain a concentration of 9 × 10^5^ cells mL^−1^. This allowed us to match performance between the runs and to verify the hypothesis that a more tailored control of feed could improve upon the manually triggered version.

The results showed promise in maintaining stable cell concentrations, with the fully automated version naturally producing a more stable concentration due to the limitations of the manual feed. Notably the noise and spikes previously observed during CART batch 2 was less pronounced in these runs suggesting it may have been due to the first usage of the new probes.

In both cases, cell growth continued for a brief period after cessation of the feeds, prior to a decline phase. The decline was more drastic in the manual variant of the experiment, but both indicated the initiation of the phase with drops in capacitance values. Notably, capacitance dropped prior to manual viability measurements detecting cell viability losses, a phenomenon which has been observed before in other cell types [42]. This earlier drop in capacitance was attributed to both cell shrinkage during latter stages of culture, and the discrepancy between dye permeation and charge escape in partially broken membranes.

This observation is important as it demonstrates how capacitance measurements detect membrane integrity reductions earlier than traditional nuclear staining‐based assays for cell counting. In nuclear staining a large dye molecule must pass the cell membrane. Intact cells exclude the dye and thus do not get stained thus allowing counting of total and stained/unstained cells. However, if a cell is in the preliminary stages of dying, membrane integrity may be reduced insufficiently to permit the dye to pass. In contrast, capacitance is based on the ability of the cell to hold charge, and even with the smallest breakages in membrane integrity this ability is lost by cells, thus allowing detection of death‐phase initiation to be captured far in advance of manual sampling (especially when considering manual sampling from a practical perspective is limited to once or twice a day and thus is also a function of operator schedule). As CGT products need to be optimally functional to be administered into a patient, having methods which show the initiation of decline earlier will reduce the numbers of ineffective cells which are administered into patients, which in turn may reduce dose size requirements proportionally.

### 3.5 CAR‐T Cell Bioreactor Automated Control Studies

**FIGURE 8:**
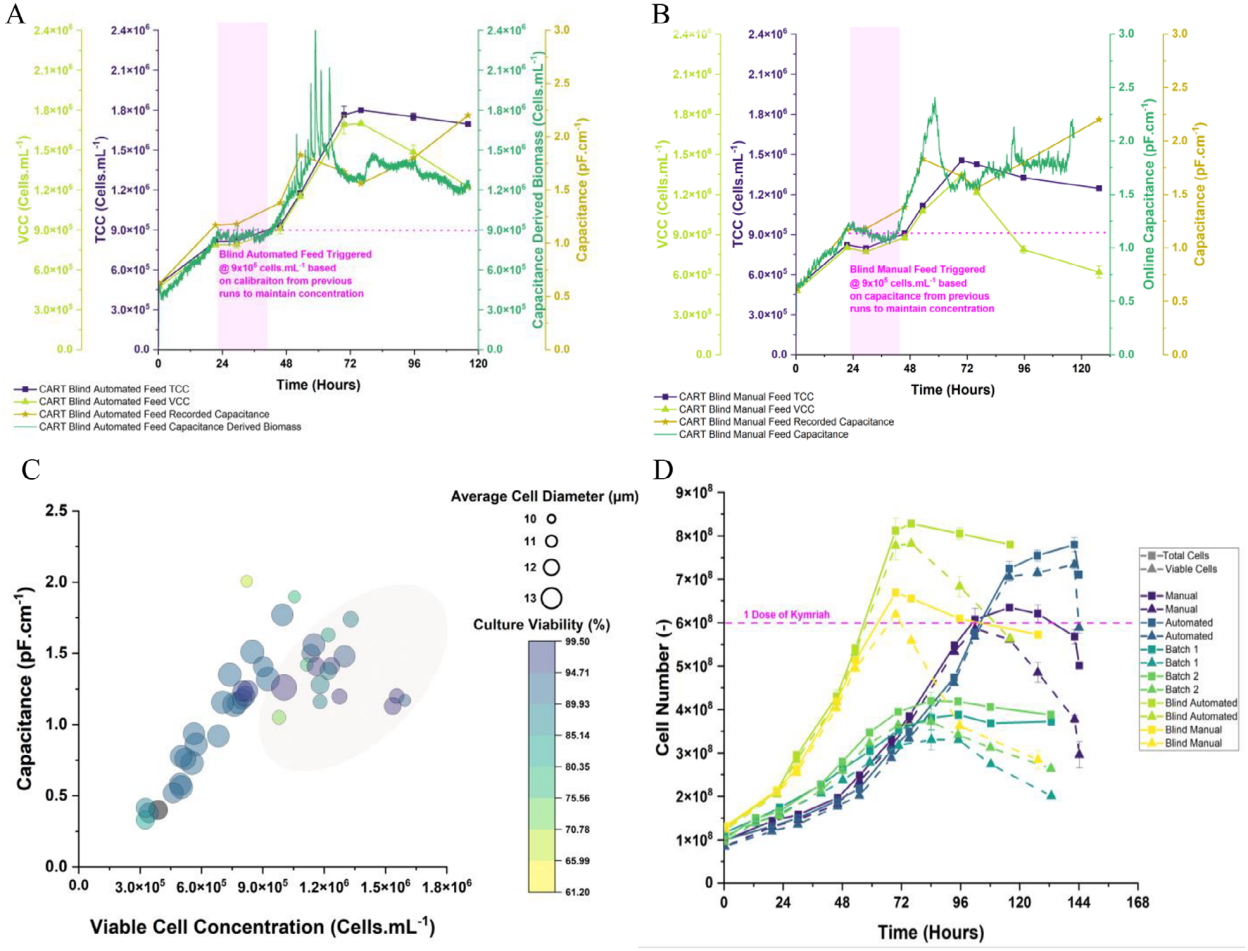
Summary of automated CAR‐T cell runs. Panels (A and B) show the culture outputs for the blind automated and manual feeds, respectively. These runs were performed based on only using historic data values to trigger feeds and feed rates. Panel (C) shows a bubble‐plot for all of the presented CAR‐T cell data, with the highlighted region once more depicting how the linear trend skews with late‐stage culture. Finally, Panel (D) shows the comparison of cell numbers obtained throughout the set of CAR‐T cell runs. Marked on the graph is a cutoff for a dose of Kymriah.

Having now generated a historic data set for the calibrated automation and the manual control, we then proceeded to perform a blind run. Using only the values generated from the previous experiment, both manual and automated control were operated based on historic expectation and calibration (for the automated experiment). During the manual run, feeding was triggered at the capacitance value of 1.1 pF cm^−1^ which approximated 9 × 10^5^ cells mL^−1^, whilst the automated run was set to trigger at the same cell concentration, using the calibration from the previous run. These data are shown in Panels **A** and **B** of Figure 8.

Once more, we observed that no flooding or other error occurred during either feeding regime (important in a proof of concept as flooding the bioreactor would result in run failure). No adverse noise related events were observed during the automated run either, suggesting brief fluctuations in signal pose minimal risk, although this tolerance will need to be evaluated to a greater extent in future development studies. Notably, adjustments to signal filtering can already be performed if this presents as issue.

Additionally, we observed that cells grew with improved exponential rates prior to the inevitable decline phase. This resulted in an increased cell yield compared to the batch runs (ca. 1.5 × 10^6^ cells mL^−1^) and was comparable to the previous results where maxima of around 1.8 × 10^6^ cells mL^−1^ were observed, suggesting the methods are reproducible and improve culture outputs.

For completeness, we include Panel **C** which demonstrates the same tight, linear correlation between viable cell concentration and capacitance measurements that was observed in CHO and T cells. Once more the deviation of this trend is only noticeable during the latter stages of culture where we observe cell shrinkage and reductions in population viability, which reiterates the applicability of capacitance as a potential addition to harvest point analytics.

Finally, the figure shows how the collective CAR‐T cell runs perform in terms of absolute cell yields, demonstrating clearly how a limited batch process that failed to achieve a dose of product can be quickly improved to create an excess of material (Panel **D**). For example, we observed that compared to the batch (ca. 4 × 10^8^ cells), the (blind) manual and automated processes achieved yields of 6–8 × 10^8^ viable cells (or 150%–200% improvements). Whilst feeding will obviously improve cell yield, the result demonstrates that capacitance can successfully automate and optimize such strategies.

## 4 Discussion

Capacitance technology has been a stalwart tool in biopharmaceutical manufacturing for many years over a wide variety of applications [18, 20, 24, 42, 43, 44, 45, 46, 47, 48, 49, 50, 51]. Its adoption in cell and gene therapy manufacturing is, however, limited.

This is because CGT processes are currently limited in scale and often employ single‐use technologies which renders the format of expensive, reusable probes prohibitive. However, there is a current drive to translate these processes into bioreactors. To align with this, Aber Instruments has begun focusing on sensors that are suitable for the smaller‐scale bioreactor formats (100–500 mL, high‐throughput bioreactor types such as AMBRs, DASBOX, and Applikon MiniBio systems, for example) that could be used in autologous processes—the Pico probe used in this study is one such example. Our aim was to show how the technology employed in these beta probes is not only representative of the larger, reusable probes, but can be applied to improve culture processes for CAR‐T cell production as a case study for use in the wider CGT field.

Our first experiments highlighted the previously established Futura probes in cultures which closely resemble current use. For this we performed a triplicate of CHO cell cultures in 1L BioBlu 1c single‐use reactors. The signal values were high, ranging from 1 to 30 pF cm^−1^ due to the higher density of CHO cultures compared to CGT processes. These signals have also been used to derive parameters to inform changes to culture health, however they are beyond the scope of this application. For deeper understanding of these metrics we refer you to more appropriate literature [52, 53].

When compared to the Jurkat cultures we observed that signals were far lower than those in CHO cultures with a range of 1–3 pF cm^−1^. This was expected due to the smaller size of T‐cells compared to CHO cells and a lower overall concentration achievable, of which both factors influence capacitance signal magnitude. Whilst this range was still operable for determining the concentration of viable biomass within the bioreactors, we note that derivation of the additional parameters for increased information of the culture is not advised at such low values. Thus whilst the probes hold the potential for biomass monitoring, cell density estimation and feed/harvest triggering, the additional benefits obtainable from parameter derivations are currently not possible. However, this opens potential lines of future investigation to optimize these low range measurements and signal amplification/sensitivity of the probes themselves.

During the transition from CHO to Jurkat processes we made an irregular shift in bioreactors as the single‐use BioBlu 1c vessels from Eppendorf were not obtainable due to pandemic‐related shortages. However, despite the larger vessel size (now a 2L glass vessel), we found that results remained proportional, and trends were conserved. This evidenced the ability of the probes to maintain continuity across varying scales and formats.

During the Jurkat experiments we focused on using capacitance and capacitance‐based measurements to incrementally improve feeding strategies in line with methodologies which could later be automated. The limitations in such experiments however were based on the fact that no automation was available at the time, with the primary author instead acting as unbiased controller. Despite this, we found that the strong correlation between cell concentration and capacitance output during the early phases of the bioreactor processes allowed us to predict when to trigger the respective feeds. Whilst unidyllic in real life, the implication that an operator can rapidly obtain methods to predict and schedule feeding naturally acts as a precursor to models which may be able to perform the same function automatically. Ideally, direct integration of probe to system for closed‐loop feedback would be implemented.

The larger scale of the Jurkat study also skews the context to the improvements to the culture outputs. Within the summary table in Figure 5 we note that the batch run creates 2.9 doses of cells (assuming dose sizes for the treatment of B‐cell lymphoma), which seemingly makes the improvements seem gratuitous. However, starting cell yield and quality in CGT applications is far lower than an immortal Jurkat line. Subsequently, it would be improbable to think CAR‐T cell expansion could produce enough to seed a 2L vessel, especially when considering the limited proliferative capacity of such cells. Thus whilst the results are descriptive of the magnitude of improvement, the results must be taken with such contextualization in mind, hence the inclusion of the doses per L metric to the table. This context can be further coupled with the final automated processes which demarcate the one dose cut‐off on the final comparison between cell yields.

Having processes which can improve from an average of 2 doses per liter to 3–3.7 doses per liter (using the B‐cell lymphoma doses as an example) would allow patients to potentially have a single biopsy used for multiple doses of product which can be cryopreserved. Such improvements would extend processing by 20–30 h but would potentially negate the need for a second biopsy, and subsequent reprocessing and re‐isolation of T cells, transformation into CAR‐T cells and expansion prior to reinjection. This would ease the burden on scheduling of CAR‐T cell bioprocessing considerably and allow for cheaper, more efficient production, especially when considering this was only a proof‐of‐concept study and further optimization would naturally be performed before implementation and technological acceptance. However, there is a limitation to our assessment of doses recovered which must also be discussed. Our experiment assumed that all cells reached after capacitance‐based controls would be identical to the quality of the cells in standard processes, and that 100% of the Jurkat cells would translate to 100% of transduction efficiency in an equivalent CAR‐T cell process. While we also assumed this 100% product for the baseline batches for comparison, the need to test for these product quality attributes would be required prior to determination of the exact improvement (or otherwise) to dose yields.

During the CAR‐T cell experiments we performed analyses on cell yields and viability to assess the potential functions of the probes themselves, however we recognize that future studies will need to incorporate more information on the maintenance of cell identity and function by way of biomarker expression, exhaustion and potency assays. Such outstanding questions arising from this work include: does the higher rate of feeding contribute to changes in metabolite production, and do the cells tend toward exhaustion faster than current processes? Another important area which should be further investigated is how the late‐stage changes to capacitance correlate to not only viable cell mass/shrinkage, but to the functional outputs of the cells themselves. Furthermore, deeper understanding of the process changes at the latter stages could also allow for the automation/fine tuning of additional control strategies beyond harvest to involve feeding adjustments or the addition of other process related boli such as Dynabeads or cytokines.

Through this study, we also observed cell population shrinkage (measured alongside offline viability/concentration and which were detectable by preceding drops in capacitance, but which may also be influenced by other factors such as activation levels of T‐cell populations). By expanding on this study in the manner discussed previously, ideal harvest points may be able to be determined and subsequently automated which would be (especially) beneficial to autologous production platforms. This would require comparative testing of cells before and after these morphological changes occur to accurately gauge harvest point optima and validate culture performance using more clinically relevant and quantifiable metrics. We envisage such combinations of information could lead to improved likelihoods of manufacturing success as the live capacitance information would reflect differences in patient‐derived cell quality and of overall bioreactor performance in a predictable manner.

Finally, though we have mentioned in depth the potential benefits of capacitance technology in cell and gene therapy manufacture, we would be remiss to not address the challenges involved with its implementation. The first such limitation has been observed within this article: that CGT cell types are often smaller and grow less densely than mammalian systems used for antibody manufacture (e.g. CHO cells). This means that with current technology signals will be limited to the lowest range of a capacitance probe's capability (allowing possibly for the exception of perfusion processes). That is not to say that trends cannot be observed, and correlations drawn between signal and viable biovolume, but that deeper complexities based on cell differentiation, activation or morphological changes could also influence capacitance measurements and will likely require further investigation on a case‐by‐case basis.

The cost and format in which the technology is employed may also be a challenge which requires solving. Here we employed a semi‐reusable (i.e., sterilizable for a few reuses but not indefinitely), beta‐version probe highlighting that commercial availability and use within the CGT space of such formats remains limited at the time of publishing [54]. As CGT products are expensive and complex, the probes (especially in single use offerings) could add costs to already expensive single‐use bioreactors—especially if the probe technology was left in an unscaled format. However, the same could be said for alternative online monitoring options such as Raman spectroscopy, which—though powerful—requires sophisticated equipment and data analysis methods to use.

Cognizant of this, we still believe that these additional costs and initial complexities would be justified should the technologies enable automation (reducing manual operator intervention and workload), reduced handling errors (reducing the cost associated with batch failure), and allow CGT manufacturing processes to detect, control and improve these highly complex bioprocesses in which even apparently minor changes to conditions can create large impacts on the final product quality [55].

## 5 Conclusion

In this study, we have provided early conceptual evidence for the successful application of capacitance technology to aid in cell and gene therapy upstream process control. The technology was shown to reliably translate trend data from CHO cell culture to Jurkat and CAR‐T cell processes. We have shown that despite the signal being below the typical ranges found in wider biotechnology applications, the raw capacitance values were ultimately robust in their assessment of CGT‐relevant viable cell concentration. Additionally, we demonstrated that feeding can be automated by bioreactors using capacitance probes. This ability to reduce operator interference, and improve culture yield and efficiency, could ultimately reduce product COGs, and diminish some of the logistical challenges associated with CGT manufacture. The study may also act as a framework for a rapid workflow to improve culture feeding strategies in cases where fully manual and lab‐based techniques are used.

The findings presented in this paper warrant further investigation. Initially these methods should be combined with potency and cell identity testing to validate cell quality and identity. Assuming the cells retain their quality attributes, another line of investigation would be the development of fuller process control (e.g., automating cell harvest to ensure that the highest quality cells are routinely recovered). In addition, with capacitance serving as a viable automation method, the advancement of probes to cater to the lower signals found in CGT processes could enable derivation of capacitance‐associated parameters to unlock further levels of process control and understanding. Combined, these may even pave the way toward individualized bioprocesses for autologous medicines. Additional avenues of investigation may include the possibility to generate and model large‐scale datasets to better describe the range/tolerances in cell variability, the development of adaptive control systems, and the improvement of advanced bioprocessing options (such as capacitance‐controlled perfusion).

## Author Contributions

**Ivano Luigi Colao** performed all experiments, contributed to hypothesis generation, contributed to experimental design, conceived hypothesis of linking feeds to capacitance‐derived cell growth kinetics, analyzed the data, processed the data, created the figures, and wrote the manuscript. **Jacob Cunningham** contributed to study conception, contributed to funding acquisition, contributed to hypothesis generation and experimental design, and proofread and critiqued the manuscript. **Matthew Lee** contributed to study conception, contributed to funding acquisition, contributed to hypothesis generation, and proofread the manuscript. **Stephen Goldrick** contributed to funding acquisition, contributed to data analysis, contributed to hypothesis generation, contributed to experimental design, proofread and critiqued the manuscript, and contributed to study conception. **Qasim Ali Rafiq** contributed to funding acquisition, contributed to data analysis, contributed to hypothesis generation, contributed to experimental design, proofread and critiqued the manuscript, and contributed to study conception. All authors agree to the content of the manuscript.

## Conflicts of Interest

The study was performed during a Knowledge Transfer Partnership (KTP) between University College London and Aber Instruments Ltd. (a biotechnology company that manufactures capacitance probes). Jacob Cunningham and Matthew Lee are employed by and own shares in Aber Instruments. Aber Instruments has patents pending regarding the Futura Pico probe used to generate data. 17771561.2 EU, 16/333,795 USA. Ivano Colao, Qasim Rafiq, and Stephen Goldrick have no competing interests.

## Data Availability

The data that support the findings of this study are available from the corresponding author upon reasonable request.
